# Overqualification and Underemployment: Italian Validation of the Scale of Perceived Overqualification (SPOQ-IT) in the Nursing Profession

**DOI:** 10.1155/jonm/8165533

**Published:** 2025-03-18

**Authors:** Martina Batino, Jacopo Fiorini, Simona Frigerio, Alessandro Sili, Francesco Zaghini

**Affiliations:** ^1^Department of Biomedicine and Prevention, University of Rome Tor Vergata, Rome, Italy; ^2^Nursing Department, Policlinico Tor Vergata, Rome, Italy; ^3^Nursing Department, University Hospital City of Science and Health, Turin, Italy

**Keywords:** nurse, overeducation, overqualification, underemployment

## Abstract

**Background:** Acquired competencies, skills, and abilities can lead professionals into an overqualification situation that is associated with absenteeism, desire to abandon the respective profession, negative work performance, and interpersonal conflicts.

**Aim:** To develop and validate the Italian version of Maynard's Scale of Perceived Overqualification (SPOQ).

**Method:** The SPOQ was linguistically and culturally adapted to develop and validate an Italian version (SPOQ-IT), calculating a content validity index and testing the scale validity with a cross-validation approach using Cronbach's alpha, exploratory, and confirmatory factor analyses (CFAs).

**Results:** The SPOQ-IT had a good content validity index. Two dimensions (“overqualification” and “underemployment”) emerged from the exploratory and CFA. Age, gender, and working years affected the overqualification and underemployment perception in the Italian nursing profession.

**Conclusion:** The SPOQ-IT has good psychometric properties and demonstrated that acquired competencies, skills, and abilities are often underexploited in the Italian nursing profession, leading to overqualification and underemployment perceptions.

**Implications for Nursing Management:** Nursing managers should govern and monitor the overeducation and its effects on organizational and patients' outcomes because the implementation of acquired competencies, skills, and abilities improves work performance and the quality of nursing care and avoids underemployment condition.

## 1. Introduction

Over the period 2012–2018, due to turnover, the number of nurses in employment in Italy fell by approximately 25,000 [1]. Today, fewer nurses work in Italy than in almost all Western European countries, and their number (6.2 per 1000 citizens) is 25% lower than the European average [2]. The shortage of nurses is, of course, not confined to the Italian context, but is an international phenomenon with various causes, including, in particular, the stress and burnout to which nurses are often exposed [3], and low job satisfaction, associated with shortcomings in professional development, and in the useful application of acquired training and skills [2]. In scientific literature, this last aspect has been addressed using the term overeducation or overqualification [4–6].

When workers have greater knowledge, skills, and abilities than those effectively required to carry out their jobs, they may be considered or perceive themselves to be overeducated or overqualified [5, 6]. A salient example of the emergence of this phenomenon occurred in the last century, when a significant rise in demand for increasingly qualified personnel [4, 5] gradually led to an exponential growth in training and the acquisition of skills, without a contemporaneous continuation in the evolution of labor demand [7–9], therefore indirectly leading to a rise in the perception of overqualification [6]. The phenomenon has become widespread throughout the world, and various scientific studies have directly addressed it [10–12]. In the healthcare context, in particular, the overqualification of nurses is an international and rapidly growing phenomenon [13].

It has been estimated that the share of overqualified nurses in Italy is approximately 23% and 13% three and five years, respectively, after completing their nursing qualification [14]. Overqualification can have various negative repercussions on individuals, in terms of stress, the development of health disorders, and job satisfaction [6, 15], and on organizations, in terms of higher rates of absenteeism, turnover [6] and reduced performance [15, 16], and interpersonal conflicts [17].

Given the importance and relevance of this phenomenon, various tools have been used to measure overqualification and its evolution over time, including the Perceived Overqualification Scale (POS) [18], the Subjective Underemployment Scale (SUS) [19], and the Scale of Perceived Overqualification (SPOQ) [6]. Among these scales, the SPOQ is that which has most readily been adapted and applied to the nursing profession. Indeed, multiple studies have been conducted using this tool in the nursing field, in various countries, and have highlighted how overqualified nurses tend to perceive higher levels of stress and be at increased risk of developing burnout [20] and experience lower job satisfaction [21, 22]. Furthermore, according to its authors, the SPOQ is suitable for investigating both objective aspects, in terms of the effective surplus of education, and subjective aspects, such as measuring the perceptions that nurses have regarding their employment, for example, “I have more skills than those required by my job” and “Even with less training or experience, I could do my job just as well.” Finally, validation of the SPOQ, in the Anglo–Saxon context, has demonstrated its high internal consistency, reliability, and good construct validity [6]. Indeed, in its first validation, the instrument achieved an internal consistency of *α* = 0.92, a test–retest reliability of *r* = 0.89, and good construct validity (*F*[2, 222] = 4.54, *p* < 0.05, n2 = 0.04), with a nine-item composition, and a Likert response scale of 1–7 (1 = completely disagree and 7 = completely agree) [6].

Considering the global importance of overqualification, with repercussions for both individuals [8, 23] and organizations [24], the translation and cultural adaption of the SPOQ to the Italian nursing context would allow the scientific community to measure and collect data on the construct in Italy, compare the data with that of other countries and existing literature, and investigate differences in terms of the development and recognition of the nursing profession within the wider world of work. In light of the above, this study aims to validate the SPOQ, as the most commonly used instrument for exploring the construct of overqualification in nursing, in the Italian cultural context.

## 2. Methods

The cultural adaptation of the SPOQ [6] and the validation of the resulting Italian version, the SPOQ-IT, was carried out in Italy between February and July 2022. According to the study's aim, the work was divided into 2 successive phases; first, the translation and cultural adaptation of the original scale, and second, the validation of the Italian version conducted on a sample of Italian nurses.

### 2.1. Phase 1: Translation and Cultural Adaptation

Having obtained consent from the original authors of the SPOQ, this phase involved the following individual activities: two forward translations, synthesis of the translations, back translation, content validity analysis, and face validity analysis [25].

### 2.2. Forward Translations

The SPOQ and its instructions were translated from the original language into Italian by two independent bilingual translators with expertise in clinical and educational studies. The translations were carried out to maintain the natural colloquial style of language of the original scale.

#### 2.2.1. Synthesis

The two translated versions of the scale were submitted to the research team, which evaluated the individual items to assemble a single version, resolving inconsistencies in items between the two versions through discussion, to maintain the coherence of the construct.

#### 2.2.2. Back Translation

The single translated version of the scale was then translated back into English by a native expert who had not seen the original version. The back-translated version was then compared with the original one by the research team and the two translators to verify its conceptual equivalence. At the end of this process, having verified the correspondence between the original scale and the single translated version, the latter was approved as the Italian translation (SPOQ-IT) of the original scale.

#### 2.2.3. Content Validity

To verify the content validity of the SPOQ-IT, the scale was presented to a panel of healthcare professionals, including managers, lecturers researchers, and, therefore, experts in healthcare research, training, and organization. These experts were asked to evaluate the relevance of each item of the SPOQ-IT in investigating overqualification, using a 4-step Likert scale, from 1 (not relevant) to 4 (highly relevant). To calculate the item content validity index (I-CVI) and the scale content validity index (S-CVI), the responses were dichotomized into two categories, “relevant” and “not relevant,” by combining values 3 and 4 and values 2 and 1. The I-CVI was, therefore, calculated as the number of experts who were assigned “relevant” to each item, divided by the total number of experts. The sum of I-CVI was then divided by the total number of items to determine the S-CVI [26].

#### 2.2.4. Face Validity

To evaluate face validity, a purposive sample of clinical nurses and researchers was recruited to achieve a heterogeneity of clinical work settings, professional expertise, and seniority in terms of work years. The respondents were asked to express in a dichotomous manner (YES/NO) the level of comprehensibility, clarity, and readability of each SPOQ-IT item and were offered the possibility to add any comments [27].

### 2.3. Phase 2-Validation of the SPOQ-IT

The second phase of the study involved the validation of the translated instrument and the measurement of its psychometric characteristics. The SPOQ-IT was, therefore, administered in the paper form to a convenience sample of Italian nurses, working in 3 hospitals and 30 different wards, in 3 locations, one in Northern Italy, one in Central Italy, and one in Southern Italy. Nurses involved in direct clinical care of patients, shift workers, and others on fixed-term or permanent contracts were included. However, nursing coordinators and managers were excluded from the sample. The time taken to complete the questionnaire containing the SPOQ-IT varied from 15 to 20 min. To guarantee the privacy and anonymity of participants, a box was set up in each ward so that the participants could anonymously drop off their completed questionnaires. In addition to the SPOQ-IT, the questionnaire included a section for the collection of data on sociodemographic variables (age, gender, and civil status) and work variables (seniority in terms of working years, qualification level, daily work hours, weekly overtime hours, and number of work absences over the last 6 months).

### 2.4. Statistical Analysis

The sociodemographic and work variables were analyzed using descriptive statistics. To verify the distribution of the items in the Italian version of the SPOQ-IT, the values of mean, standard deviation (SD), skewness, and kurtosis were calculated. The validity of the scale was tested using the cross-validation approach [28] by randomly dividing the sample into two subsamples. The first subsample was used to conduct exploratory factor analysis (EFA), while the second subsample was used for confirmatory factor analysis (CFA). Following the guidelines of Kyriazos [29]; a sample size of at least 200 participants was deemed adequate for both EFA and CFA. In the EFA, the dimensionality of the scale was verified using the maximum likelihood (ML) method and promax rotation. The number of factors to extract was decided based on the simplicity of the solution (factor loadings > 0.30, with no cross-loadings), the examination of eigenvalues greater than 1, the interpretability of the factor structure [30], and the theory of the meaning of dimensions [31]. Subsequently, on the second subsample, CFA was performed with the ML estimation method to confirm the dimensionality of the scale. To evaluate the adequacy of the measurement model, the following fit indices were considered: the chi-squared test (*χ*^2^, not significant), the root mean square error of approximation (RMSEA, < 0.06), the comparative fit index (CFI, > 0.90), the Tucker–Lewis index (TLI, > 0.90), and the standardized root mean squared residual (SRMR, < 0.08) [32]. Having verified the dimensionality of the SPOQ-IT scale, the internal consistency of each factor was examined using Cronbach's alpha coefficient (α), considering values ≥ 0.70 as sufficiently reliable [33]. The values relating to the dimensions of the SPOQ-IT were calculated using the average. Correlations were used to examine associations between the dimensions of the SPOQ-IT and the nurses' sociodemographic and work variables. For continuous variables such as age, Pearson's correlation coefficient (r) was used; for categorical variables, such as gender, the point-biserial correlation coefficient was used; and for ordinal variables, such as qualification level, Spearman's correlation coefficient (*ρ*) was used. For the analyses regarding descriptive statistics, correlations and the EFA, the statistical package SPSS® Version 22 was used, while the CFA was conducted using the package MPlus® Version 7.1. The significance level was set at *p* < 0.05.

### 2.5. Ethical Considerations

The study has been approved by the Ethics Committee of the University Hospital of Rome Tor Vergata, where the study was conceptualized (Prot. no. RS143.21) and then authorized by each hospital and was conducted according to the principles of the Declaration of Helsinki [34]. All voluntarily enrolled participants were provided with information regarding the purpose, objective, and data collection methodology of the study. The collected data were analyzed with a methodology to ensure the anonymity of individual participants.

## 3. Results

### 3.1. Phase 1

For the face validity analysis, 14 clinical nurses and researchers were enrolled and declared that the scale items were understandable, clear, and legible but suggested slight modifications to two of the items to improve their comprehensibility. As a result, Item 1 was modified from “My job requires less education than I have” to “My job requires less training than I have” and Item 7 was modified from “My training level is higher than the level required by my job” to “My training level is higher than that required by my job.”

The Italian version of the scale (SPOQ-IT) achieved a S-CVI of 0.87. All items achieved an I-CVI of ≥ 0.78.

### 3.2. Phase 2

#### 3.2.1. Sociodemographic and Work Variables

A total of 500 nurses were invited to participate in the validation study, and 56.6% took part (Table 1). The final sample was therefore composed of 283 nurses, of which 82.7% (*N* = 234) were female, having an average age of 39.9 years (SD = 11.1), and having worked on average for 16.4 years (SD = 11.8).

#### 3.2.2. Item Characteristics and EFA

**Figure 1:**
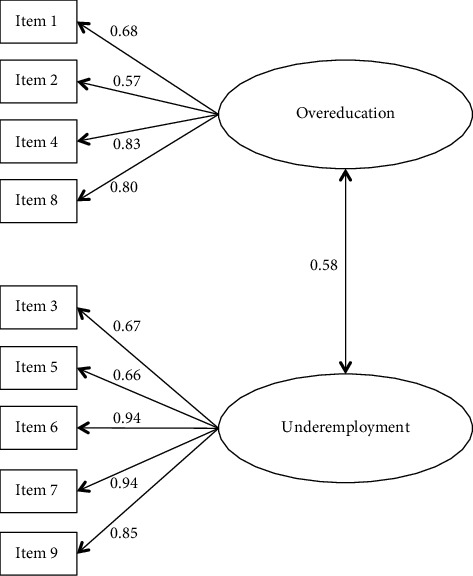
Confirmatory factor analysis of the SPOQ-IT.

The results of the analysis of the distribution of the SPOQ-IT items (Figure 1) indicated that all the items fell within the −1 to +1 range, allowing the values of the skewness and kurtosis indices to be considered acceptable [35, 36]. Furthermore, the range of mean responses varied between 1.93 (SD = 1.17) and 2.95 (SD = 1.54). The EFA, evaluated for 147 nurses, indicated that the 2-factor solution of the scale was the most satisfactory (Table 2). The factor saturations were all high and significant (*p* < 0.001). The saturations of all items were greater than 0.555, confirming that all items were good indicators of the corresponding dimension [37]. The dimensions identified in the analyses, regarding the content of the individual items composing them, were evaluated by the panel of experts, who decided to rename them as follows: overqualification, as a term referring to the surplus of training (5 items), and underemployment, as a term referring to the underuse of skills (4 items).

The model fit indices were *χ*^2^ (26, *N* = 146) = 69.271, *p* < 0.001; RMSEA = 0.106 (90% CI = 0.076–0.137), *p* value (RMSEA < 0.05) 0.002; CFI = 0.947; TLI = 0.927; and SRMR = 0.047. All factor saturations were significant (*p* < 0.001) and greater than 0.57, confirming that all items were good indicators of the corresponding dimension. Finally, as expected, the two dimensions that emerged from the analyses (overqualification and underemployment) correlated with each other (*r* = 0.58; *p* < 0.001).

#### 3.2.3. Correlation Analysis

Regarding the correlations between the dimensions of the SPOQ-IT and the sociodemographic and work variables (Table 3), the analysis revealed a negative correlation between age and the overqualification of the nurses (*r* = −0.15; *p*=0.11). As for gender, men considered themselves more overqualified (*r* = −0.14; *p*=0.02) and more underemployed (*r* = −0.13; *p*=0.03) than women did. A positive correlation was found between qualification level and the perceived overqualification of the nurses (*r* = 0.20; *p* < 0.01), indicating that nurses with higher qualification levels considered themselves more overqualified. Finally, regarding seniority in terms of work years, a negative correlation was found with overqualification (*r* = −0.19; *p* < 0.01), indicating that nurses with more years of experience were those who considered themselves less overqualified.

Regarding the dimensions of the SPOQ-IT scale, the nurses in our sample reported being “somewhat” overqualified (*M* = 2.90; SD = 1.35) and underemployed (*M* = 2.10; SD = 1.02). The two dimensions of the scale were significantly correlated with each other (*r* = 0.60; *p* < 0.01; Table 4).

The reliability of the SPOQ-IT dimensions, measured using Cronbach's alpha, was found to be 0.92 for overqualification and 0.82 for underemployment [33]. The corrected item-total correlation was in the range of 0.54–0.74 for the overqualification dimension and 0.63–0.88 for the underemployment dimension.

## 4. Discussion

This study aimed to develop and validate the Perceived Overqualification Scale in the Italian context (SPOQ-IT). The results of the validation confirm that the SPOQ-IT is a valid and reliable instrument with good psychometric characteristics. Furthermore, differently from the original scale [6], which is unidimensional, this cultural adaptation and validation study found that, in the Italian cultural context, the SPOQ-IT had 2 dimensions with excellent fit indices in the analysis model: overqualification, measuring the training surplus concerning the work context; and underemployment, measuring the underuse of professional skills.

The identification of these two dimensions via the psychometric testing of the scale is an innovative element, which brings further confirmation to the scientific community, for the original scale. In the definition of overqualification, Maynard identified two aspects of the phenomenon: objective overqualification, relating precisely to the surplus of training, and subjective overqualification, referring more to the perception of the professional than to the effective imbalance between skills possessed and skills put to use [6]. From the results of this study, it seems that the two dimensions of the SPOQ-IT represent this dual aspect of overqualification experienced by nurses. Indeed, on the one hand, the overqualification dimension investigates the level of training of nurses in their work context (e.g., Item 5: “My training is not fully used in this job”). On the other hand, the underemployment dimension investigates the skills and experience effectively required to carry out the nurses' work (e.g., Item 8: “Even with less experience, I could do my job just as well”). This difference emerged predominantly in the conducted analysis, though the two dimensions constitute two aspects of the same phenomenon. Overqualified nurses may work in contexts in which they do not apply certain skills and abilities daily, though these same skills and abilities may be required to be a healthcare professional. This subdivision, in the Italian cultural context, is easily explained by the rules regulating the profession and the work contracts of nurses. Indeed, for Italian nurses, there are no well-defined and pre-established clinical career paths, and so professionals who have gained training either formally or in the field may not, in many cases, be able to use the actual skills they possess, or if their skills are used, may perceive themselves to be underpaid for possessing those skills, and consequently feel underemployed with greater intensity than in other countries [38]. The evolution of the training of nurses in Italy has, in recent years, led to more nurses acquiring qualifications for managerial or leadership roles (through Master's degrees, research doctorates, etc.) or specialist professional skills (through specialist Master's degrees), which career paths do not yet fully recognize in the world of work.

The division that emerged from the SPOQ-IT psychometric analysis into the two dimensions, named overqualification and underemployment, was verified in the correlation analysis concerning the participants' sociodemographic and work variables. While, for overqualification, correlations emerged in almost all the variables investigated, underemployment was perceived much less, and the correlation was confirmed exclusively about gender. This result is useful and important in supporting the thesis that the educational surplus in the Italian cultural context is a multidimensional construct with different facets [27].

Regarding overqualification, we observed that younger people with a degree in nursing yet fewer years of work experience tended to be more likely to report overqualification. This result is coherent with previous research, suggesting that younger nurses look to the nursing professional not merely as an entry point into the world of work but also as a starting point for becoming protagonists in the national healthcare system of the future [39, 40]. Accordingly, the age group most subject to overqualification was between 25 and 30 years old, i.e., those most likely to be entering the world of work for the first time [41]. In parallel, a negative relationship was observed between seniority in terms of work years and the phenomenon under study. This is in agreement with the literature suggesting that overqualification falls over time [23].

Regarding gender, both dimensions of the SPOQ-IT showed an important correlation with the male gender. This result is coherent with previous research on gender differences in the nursing population [17] and may be partly explained, in Italy, by still prevailing stereotypes of women as wives and mothers, who take care of family and children [42, 43], and are less inclined to pursue work or career goals. On the other hand, the results of Ghignoni's study [41] suggested that women in Mediterranean countries, as opposed to in the Netherlands, were more at risk of developing overqualification than men. These data were supported by Fleming and Kler, who added that the phenomenon is more likely to occur in female subjects without children [44]. This latter aspect may be explained by the fact that some women with children may be more content to accept jobs for which they are overqualified, as their focus is on satisfying a good balance between work and family life.

### 4.1. Limitations

Despite the results obtained, certain limitations of this study must be considered. First, the results were derived from a convenience sample. Future research should, therefore, be conducted on representative and random samples of the reference population. A second limitation comes from the fact that the SPOQ-IT is a self-report instrument, which measures the perception of the participants, who may be burdened by other stress factors independent of the variables under study. Future studies might, therefore, integrate the SPOQ-IT within questionnaires measuring organizational wellbeing constructs [45] to compare the weights of other variables through external validity analysis. Lastly, we have not conducted further analysis of the validity and reliability of the scale (i.e., measurement invariance).

## 5. Conclusions

This study culturally adapted and validated an instrument for measuring the overqualification of nurses in Italy. The SPOQ-IT proved to be a valid, reliable instrument, with excellent psychometric properties and, therefore, represents an added value for the scientific community, as it allows us to investigate the two highly impactful and topical constructs of overqualification and underemployment in Italian nurses. Governing the overeducation should be a priority of healthcare and nursing manager and avoid that their followers acquired new skills, competencies, and knowledge that are not useful or applied in clinical practice.

### 5.1. Implications for Nursing Management

For managers of healthcare professionals, having such a tool is strategic, as it allows them to monitor the phenomenon over time. An increased focus on exploiting the acquired skills and abilities of individual professionals is bound to have important effects in terms of reducing work stress and the risk of developing burnout and improving work performance, patient outcomes, and the quality of nursing care in general. This instrument can also be a guide for mapping the follower's overeducation and studying an organizational strategy for their right allocation in clinical settings. Enhancing the core curriculum of its employees would enable nursing managers to encourage what they have acquired, improve their job performance, and consequently increase organizational outcomes in terms of reducing absenteeism, stress levels, burnout, interpersonal conflicts, and those of the patients.

## Figures and Tables

**Table 1:** Sample sociodemographic and work variables (*N* = 283).

	*N*	%	M	SD
Age			39.85	11.14
Gender				
Male	49	17.3		
Female	234	82.7		
Civil status				
Single	113	39.9		
Divorced	16	5.7		
Married	152	53.7		
Qualification level				
Regional diploma	105	37.1		
University diploma	24	8.5		
Bachelor's degree	154	54.4		
Seniority (work years)			16.43	11.78
Daily work hours			7.84	4.79
Weekly overtime hours			7.31	7.18
Number of work absences over the last 6 months			4.35	8.56

Abbreviations: M, mean; SD, standard deviation.

**Table 2:** Psychometric characteristics and the EFA loading factor of SPOQ-IT items (*N* = 147).

Item	M	SD	Skewness	Kurtosis	Overqualification	Underemployment
Item 3	2.84	1.61	1.00	0.23	**0.56**	0.25
Item 5	2.95	1.54	0.46	−0.79	**0.56**	0.21
Item 6	2.91	1.52	0.47	−0.57	**0.99**	−0.09
Item 7	2.76	1.50	0.76	0.16	**0.99**	−0.09
Item 9	2.95	1.50	0.49	−0.36	**0.90**	−0.03
Item 1	1.99	1.13	0.82	0.85	0.01	**0.60**
Item 2	2.13	1.22	1.00	0.23	−0.08	**0.74**
Item 4	1.93	1.17	0.92	0.83	−0.04	**0.91**
Item 8	2.31	1.26	0.79	−0.12	0.21	**0.59**
Percentage of explained variance					38.74%	25.58%

*Note:* The covariance matrix of items proved adequate for the factor loading (Barlet test, *χ*^2^ = 1782.012, *p* < 0.001, and KMO = 0.893), with the 2-factor structure explaining 64.32% of the total variance. Bold values identify the highest factor loadings for each item in the exploratory factor analysis (EFA). These loadings indicate the degree of association between each item and the two identified factors, overqualification and underemployment.

**Table 3:** Correlation between the SPOQ-IT dimensions and the sociodemographic and work variables.

	Overqualification	Underemployment
Age	**−0.15 **⁣^∗^	−0.05
Gender (1 = male; 2 = female)	**−0.14 **⁣^∗^	**−0.13 **⁣^∗^
Qualification level (1 = regional diploma; 2 = university diploma; 3 = bachelor's degree)	**0.20 **⁣^∗∗^	−0.01
Seniority in work years	**−0.19 **⁣^∗∗^	−0.05
Daily work hours	0.02	0.06
Weekly overtime hours	−0.06	0.01

*Note:* Bold values identify statistically significant results.

⁣^∗^*p* < 0.05.

⁣^∗∗^*p* < 0.01.

**Table 4:** Correlations and characteristics of the SPOQ-IT dimensions.

Dimension	M	SD	Skewness	Kurtosis	Cronbach's *α*	Underemployment
Overqualification	2.90	1.35	0.37	−0.60	0.92	—
Underemployment	2.10	1.02	0.94	0.26	0.82	**0.60 **⁣^∗∗^

*Note:* Bold values identify statistically significant results.

⁣^∗∗^*p* < 0.01.

## Data Availability

The data used to support the findings of this study are available on request from the corresponding author.
